# Surgical treatment of acute aortic dissection in a patient with SLE and prior antiphospholipid syndrome on therapy for over 30 years: a case report

**DOI:** 10.1186/s12872-022-02659-w

**Published:** 2022-05-13

**Authors:** Taira Yamamoto, Daisuke Endo, Akie Shimada, Satoshi Matsushita, Tohru Asai, Atsushi Amano

**Affiliations:** 1 Department of Cardiovascular Surgery, Juntendo Nerima Hospital, Takanodai 3-1-10, Nerima- Ku, Tokyo, 177-8521 Japan; 2 Department of Cardiovascular Surgery, Juntendo University, Hongo 2-1-1, Bunkyo-ku, Tokyo, 113-8421 Japan

**Keywords:** Systemic lupus erythematosus, Aortic dissection, Antiphospholipid syndrome, Plasma exchange, Bowel perforation, Modified triple therapy

## Abstract

**Background:**

In patients with systemic lupus erythematosus (SLE), lengthy treatment and long-term steroid use are the main risk factors for developing aortic aneurysms or aortic dissections. In patients with cardiac tamponade, hemodynamic collapse may lead to acute renal and hepatic failure.

**Case presentation:**

We report the successful treatment of a 55-year-old woman with SLE since the age of 21. She suddenly felt chest pain approximately 2 weeks before developing fever and vomiting and was admitted to our hospital. Initially, she had severe liver dysfunction and was admitted to the hepatology department, where treatment for fulminant hepatitis was initiated. However, computed tomography (CT) showed an acute aortic dissection (DeBakey type II) and severe bloody pericardial effusion. Therefore, we performed emergency pericardial drainage. Plasma exchange therapy was initiated as emergency aortic surgery was deemed impossible due to impaired liver function tests and coagulation. Ten days later, the patient developed peritonitis due to small bowel perforation, and laparotomy was performed for abscess drainage and perforation closure. She had received steroid pulse therapy at the age of 21. At 40 years of age, she developed deep vein thrombosis due to antiphospholipid antibodies and was prescribed prednisolone. She was ambulatory at 3 months after the onset of acute aortic dissection, and CT revealed a rapidly enlarging true aneurysm in the distal arch. We performed elective aortic surgery. Although there were no antiphospholipid antibodies, surgery could have led to a devastating antiphospholipid syndrome. Therefore, we decided to treat the patient with triple therapy. Methylprednisolone was intravenously administered intraoperatively and at 1 day postoperatively. The patient was discharged without complications after returning to her usual oral prednisolone regimen.

**Conclusions:**

The patient described herein had a systemic circulatory failure due to cardiac tamponade, accompanied by liver failure. This condition is a significant cause of death in patients with aortic dissection-associated SLE and is extremely dangerous. However, multi-specialty intervention helped the patient recover, and she has been attending the outpatient clinic. Aortic surgery requiring hypothermia in SLE patients with antiphospholipid syndrome and a history of thrombocytopenia or thrombosis requires a multi-disciplinary treatment team, including cardiac surgeons and medical experts.

## Background

Patients with systemic lupus erythematosus (SLE) on a long course of treatment with a history of hypertension, long-term steroid use, or SLE-related aortic pathological changes are at a high risk of developing aortic aneurysms or aortic dissections [1, 2]. In patients with SLE [3], sudden changes in the aorta can cause the SLE to rupture due to its vulnerability. In a state of cardiac tamponade, hemodynamic collapse and the effects of ongoing oral medications can easily lead to acute renal and hepatic failure, which may be severe. Antiphospholipid and other antibodies may cause pancytopenia and excessive thrombus formation; therefore, surgery with cardiopulmonary bypass (CPB) requires careful treatment planning [4–6]. Endovascular treatment is minimally invasive, but post-treatment endoleaks could be a problem [7]; conventional graft replacement is still preferred.

Approximately 10–20% of patients with SLE have antiphospholipid antibody syndrome (APS), and approximately 40% of them are positive for antiphospholipid antibodies. However, less than 40% develop thrombosis [8]. Additionally, the Japan Intractable Disease Information Center reported that approximately 60,000 patients have SLE [9]. Thus, there could be as many as 6,000 to 12,000 secondary patients with APS in Japan. Deep vein thrombosis, cerebral infarction, and habitual miscarriage are the most common complications of APS; however, some patients may also suffer from aortic dissection. Perioperative catastrophic antiphospholipid syndrome (CAPS) is the most avoidable complication; however, its exact pathogenesis and treatment must be understood.

Lupus mesenteric vasculitis is an uncommon but severe complication occurring in patients with SLE. It requires careful attention in the preoperative cardiovascular surgical field and in patients with implanted artificial valves and grafts. Furthermore, even with steroids and immunosuppression therapy, abscesses, sepsis, and systemic infections may arise as complications due to decreased immunity. Therefore, in patients with a long history of SLE treatment, various systemic complications may occur, and multidisciplinary medical intervention should be considered.

We herein report the case of a patient with SLE on medication for over 30 years who developed acute cardiac tamponade due to acute aortic dissection accompanied by several complications.

## Case presentation

A 55-year-old woman was diagnosed with SLE at the age of 21, and was hospitalized for 3 months for steroid pulse therapy. Subsequently, she continued to take prednisolone (5–15 mg/day every alternate day) from the age of 21 until now. Moreover, she had undergone surgery for necrosis of the right femoral head at the age of 30 and developed deep vein thrombosis with a positive antiphospholipid antibody at the age of 40, diabetes at 47, and cataract at 52. Her oral medications included prednisolone (5–15 mg/day every alternate day), carvedilol (1.25 mg/day), furosemide (40 mg/day), spironolactone (25 mg/day), and vildagliptin (50 mg/day). She has been hypertensive for 10 years and has been on an antihypertensive. (irbesartan, 100 mg/day). Her blood pressure at regular outpatient visits ranged from 130/80 mmHg to 150/90 mmHg.

She suddenly experienced chest pain approximately 2 weeks prior to visiting our institution. She continued to rest at home but developed fever and had several episodes of vomiting and general malaise. Upon visiting her local doctor, she was diagnosed with severe liver dysfunction and was immediately rushed to our hospital. She was initially admitted to the hepatology department, and treatment for fulminant hepatitis was started.

Physical examination revealed a height and weight of 150.5 cm and 37.8 kg, respectively. Her blood pressure was 80/60 mmHg, and her heart rate was 110 bpm in sinus rhythm. She had a fever with body temperature of 38 °C. Her extremities were cold, and her jugular veins were raging. Laboratory data were as follows: hemoglobin, 9.1 g/dL; platelet count, 182 × 10^9^/L; total protein, 5.4 mg/dL; albumin, 2.7 mg/dL; total bilirubin, 3.42 mg/dL; direct bilirubin, 2.34 mg/dL; aspartate aminotransferase, 1565 mg/dL; alanine aminotransferase, 2543 IU/L; prothrombin time-international normalized ratio, 11.39; activated partial thromboplastin time, 66.4 s; serum-creatinine, 1.85 mg/dL; hemoglobin A1c, 6.4%; brain natriuretic peptide, 452 pg/L; C-reactive protein, 6.4 mg/L; fibrin-fibrinogen degradation product (FDP) D-dimer, 34.1 µg/mL; anti-nuclear antibody, 160 H (Homogenous 160 H, Speckled 160 H); 50% hemolytic complement activity (CH50), 21; C3, 55 mg/dL; C4, 21 mg/dL; anti-cardiolipin immunoglobulin G, < 8 U/mL; direct coombs test, positive; indirect coombs test, negative; and antiplatelet antibody test, negative. Her antiphospholipid antibody test was negative after steroid treatment.

**Fig. 1:**
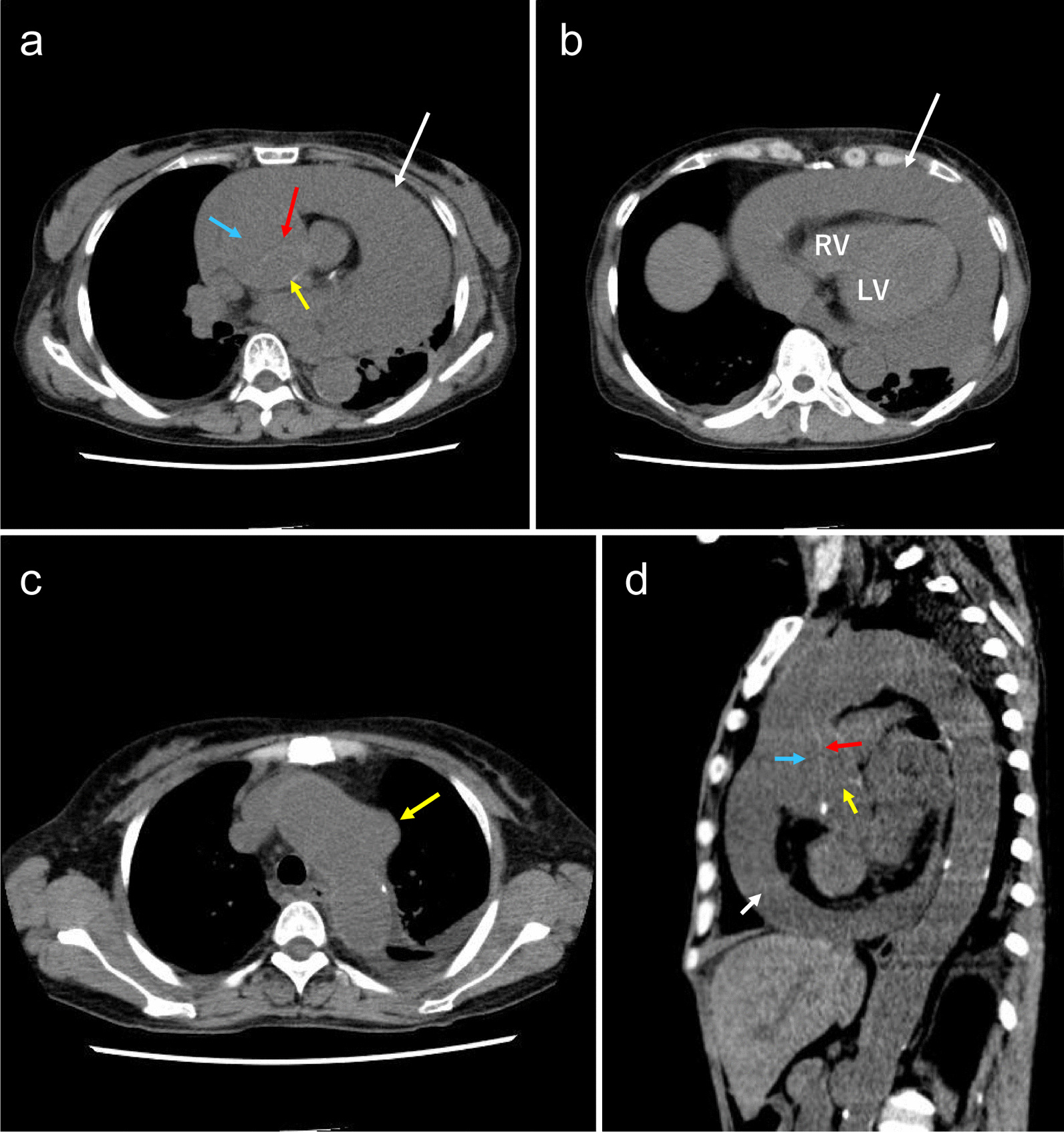
**a) **The white arrow indicates pericardial effusion, the blue arrow indicates false lumen, and the yellow arrow indicates the true lumen in the ascending aorta. The red arrow indicates the intimal flap. (**b**) The white arrow indicates pericardial effusion, and the right ventricle is collapsed due to pericardial effusion. *RV* right ventricle; *LV* left ventricle. (**c**) The yellow arrow is the true saccular aneurysm in the distal arch, and there is no dissection from the arch to the descending aorta. (**d**) Sagittal image: The white arrow indicates pericardial effusion, the blue arrow indicates false lumen, and the yellow arrow indicates the true lumen in the ascending aorta. The red arrow indicates the intimal flap

Computed tomography (CT) revealed DeBakey type II acute aortic dissection, a large amount of bloody pericardial fluid and a saccular aneurysm of the distal arch  **(**Fig. 1a, b, c and d**)**. CT showed a 50-mm-diameter pericardial effusion around the left ventricle, a 52-mm-diameter dissected ascending aorta, a 48-mm-diameter distal arch saccular mass, and a 24-mm-diameter inferior vena cava. Emergency pericardial drainage was performed, and 700 mL of bloody pericardial effusion was removed. Since her liver function and coagulation function were failing, emergency surgery was deemed impossible, and plasma exchange therapy was initiated. Her liver function gradually improved (Table 1), and she resumed eating after 1 week.

**Table 1:** Changes in tests after hospitalization

	Day 0 (at admission)	Day 0 (10 h later)	Day 1	Day 2	Day 4
Blood pressure (mmHg)	80/60	102/60	102/60	108/70	118/74
Heart rate (bpm)	110	78	78	60	90
White blood cells (X 10^9^/L)	11.5	12.2	12.7	10.1	8.8
PT-INR	11.29	2.86	1.32	1.27	1.26
aPTT (s)	66.4	54.1	62.7	37.8	44.1
AST (U/L)	1565	951	251	89	35
ALT (U/L)	2543	1940	574	222	130
Total bilirubin (mg/dL)	3.42	2.79	2.43	2.86	2.97
Direct bilirubin (mg/dL)	2.34	1.81	0.76	0.56	0.95
Total protein (g/dL)	5.4	4.8	5.1	5.2	5.1
Albumin (g/dL)	2.7	2.4	2.8	3.1	2.9
C-reactive protein (mg/dL)	5.9	5.1	1.9	4.9	17.9

*AST* aspartate aminotransferase, *ALT* alanine aminotransferase, *PT-INR* prothrombin time-international normalized ratio, *aPTT* activated partial thromboplastin time

**Fig. 2:**
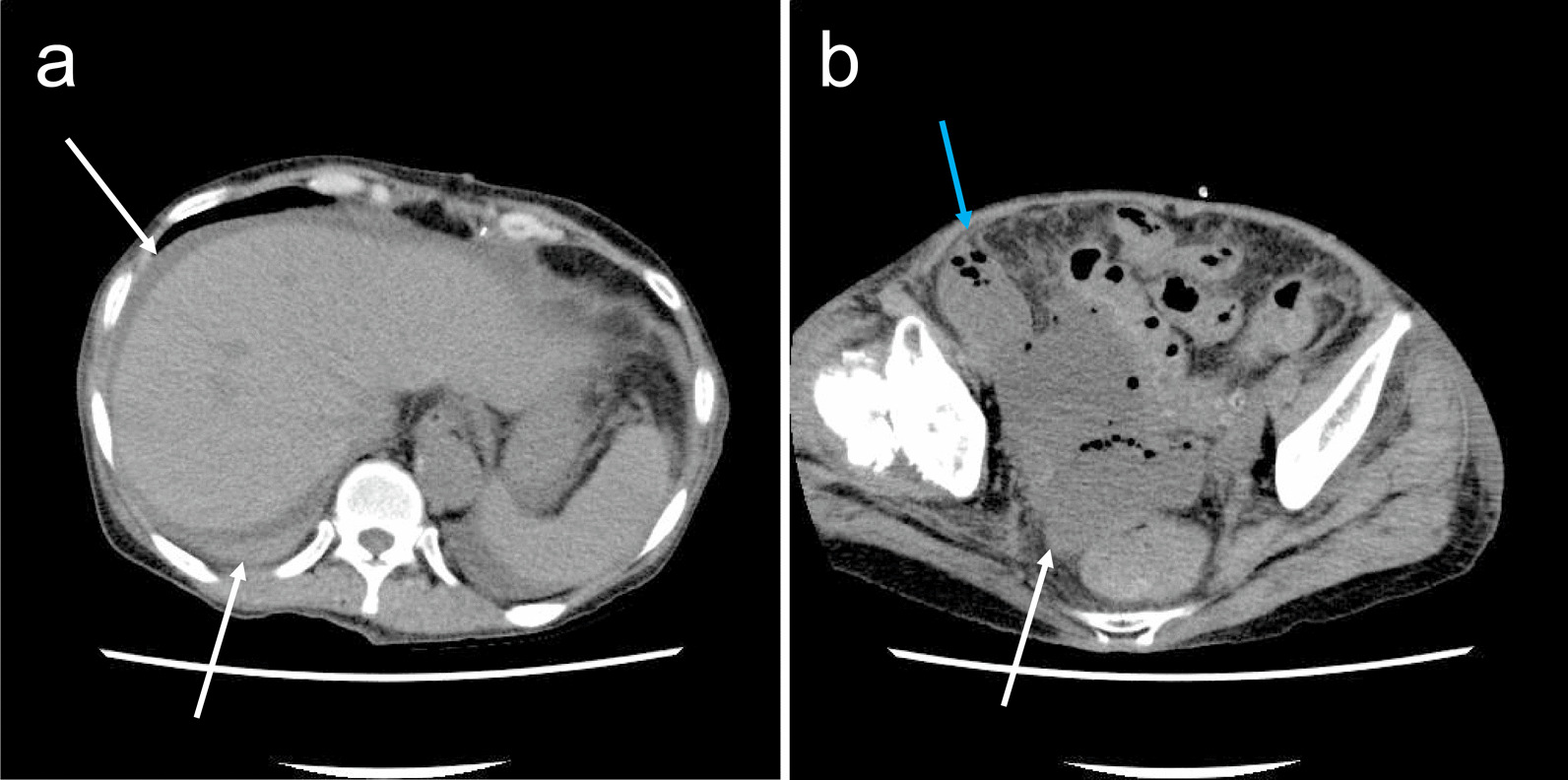
**a**) The white arrow indicates ascites around the liver. (**b**) The white arrow indicates ascites in the pelvic cavity, and the blue arrows indicate free air near the ileocecal region in the abdominal cavity

Unfortunately, she developed peritonitis due to perforation of the small intestine at 3 days after she started eating (Fig. 2a and b). CT revealed ascites in the pelvis and around the liver. The wall of the ileocecal region was thickened, and there was also a small free air bubble near the intestine. The gastrointestinal surgical team drained the foul-smelling abscess, and bacteriological examination revealed the presence of *Escherichia coli*. The surgical team subsequently opened the abdomen and performed an ileal resection and pelvic drainage.

**Fig. 3:**
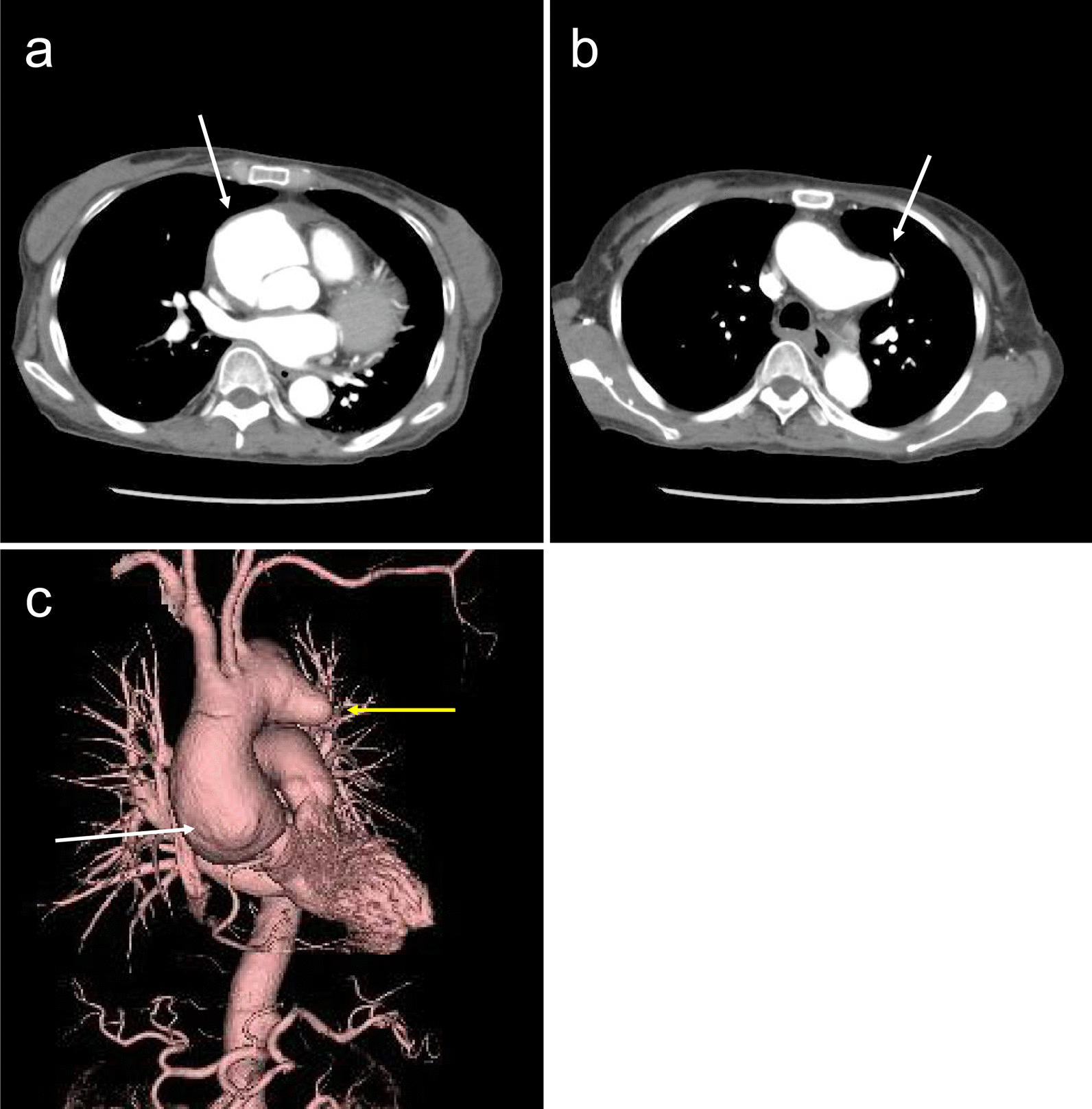
**a**) The white arrow indicates aortic dissection in the ascending aorta and no dissection in the descending aorta (DeBakey type II). (**b**) The white arrow indicates the true saccular aneurysm, which is enlarged compared to that in Fig. 1b. (**c**) The white arrow indicates the dilated ascending aorta with dissection, and the yellow arrow indicates the saccular aneurysm in the distal arch

At 3 months after the onset of acute aortic dissection, the patient’s condition improved to the point where she could walk after diligently following the treatment protocol. Three more months later, CT revealed that the diameter of the type II dissected ascending aorta had enlarged to 64 × 66 mm, and the saccular aneurysm in the distal arch had also enlarged to 56 mm. Therefore, surgery was decided (Fig. 3a–c). Although the antiphospholipid antibody test was negative, CAPS with surgery remained possible. We, therefore, developed a strict surgical plan, called modified triple therapy. In our department, patients with SLE with a history of positive antiphospholipid antibodies or thrombocytopenia are treated in accordance with the protocol for CAPS [8]. These patients are treated with intravenous gamma globulin (IVIG) for 5 days before surgery, plasma exchange during cardiopulmonary resuscitation, and IVIG for another 5 days until the FDP decreases. The postoperative platelet count begins to increase thereafter. In our patient, we calculated the predicted plasma volume [= 0.065 × body weight (kg)] × (1−Ht) and replaced 30% of that volume using fresh frozen plasma (FFP). Globulin was also administered intravenously for 5 days (200 mg/kg/day).

**Fig. 4:**
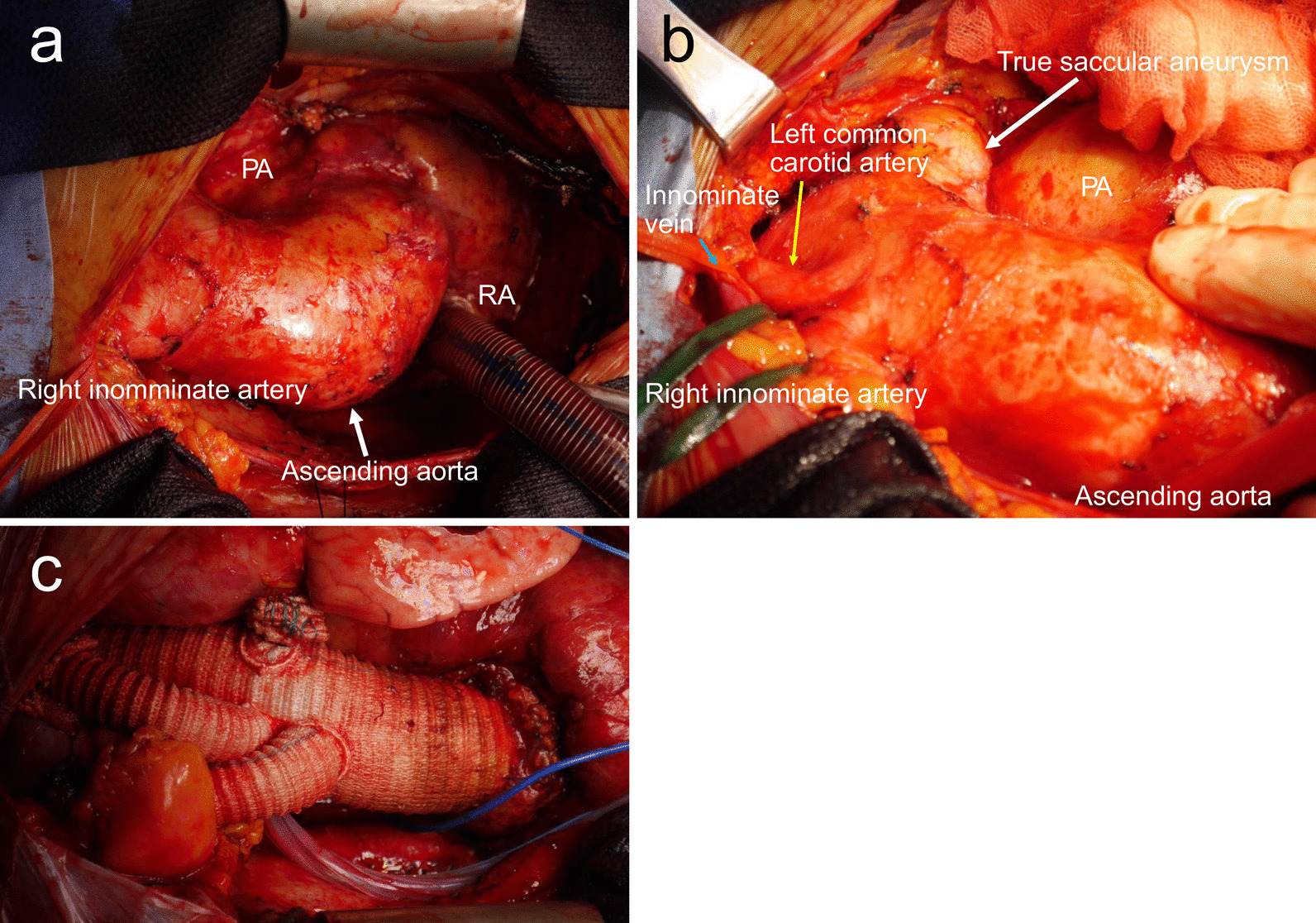
**a**) The white arrow represents the ascending aorta. *RA* right atrium; *RV* right ventricle; and *PA* pulmonary artery. (**b**) The yellow arrow represents the left common carotid artery, and the blue arrow represents the innominate vein RA, right atrium; RV, right ventricle; and PA, pulmonary artery. (**c**) Total arch replacement

Subsequently, sternotomy was performed. We established a CPB into the left atrial venting tube via the right upper pulmonary vein, inflowing from the right subclavian artery and outflowing through the right atrial appendage. We exfoliated the tissue surrounding the ascending aorta and placed a tape around the distal ascending aorta. The dissected ascending aortic aneurysm had enlarged to 65 mm in diameter. However, there was little adhesion to the right ventricle, right atrial appendage, or pulmonary artery. We also identified a saccular aneurysm of the distal arch, which had expanded to 55 mm; however, there were only a few adhesions to the surrounding tissues, such as the left recurrent nerve (Fig. 4a and b). We performed extended aortic arch replacement, removing the distal arch true aneurysm under systemic hypothermia with selective antegrade cerebral perfusion (Fig. 4c). To sufficiently protect the brain, the target minimum core temperature was adjusted to approximately 28 °C. Systemic rewarming was initiated after distal anastomosis. We performed anastomosis to the proximal aorta with double felts at the ST-junction. Pump weaning was achieved without inotropic support, and hemostasis was established in the usual manner. Circulatory arrest time was 38 min, and the aortic cross-clamp time was 98 min. The time required for CPB was 196 min, and the operative time was 6 h and 13 min. Intravenous methylprednisolone was administered intraoperatively and 1 day postoperatively. The usual dose of oral prednisolone was resumed on the second postoperative day.

**Fig. 5:**
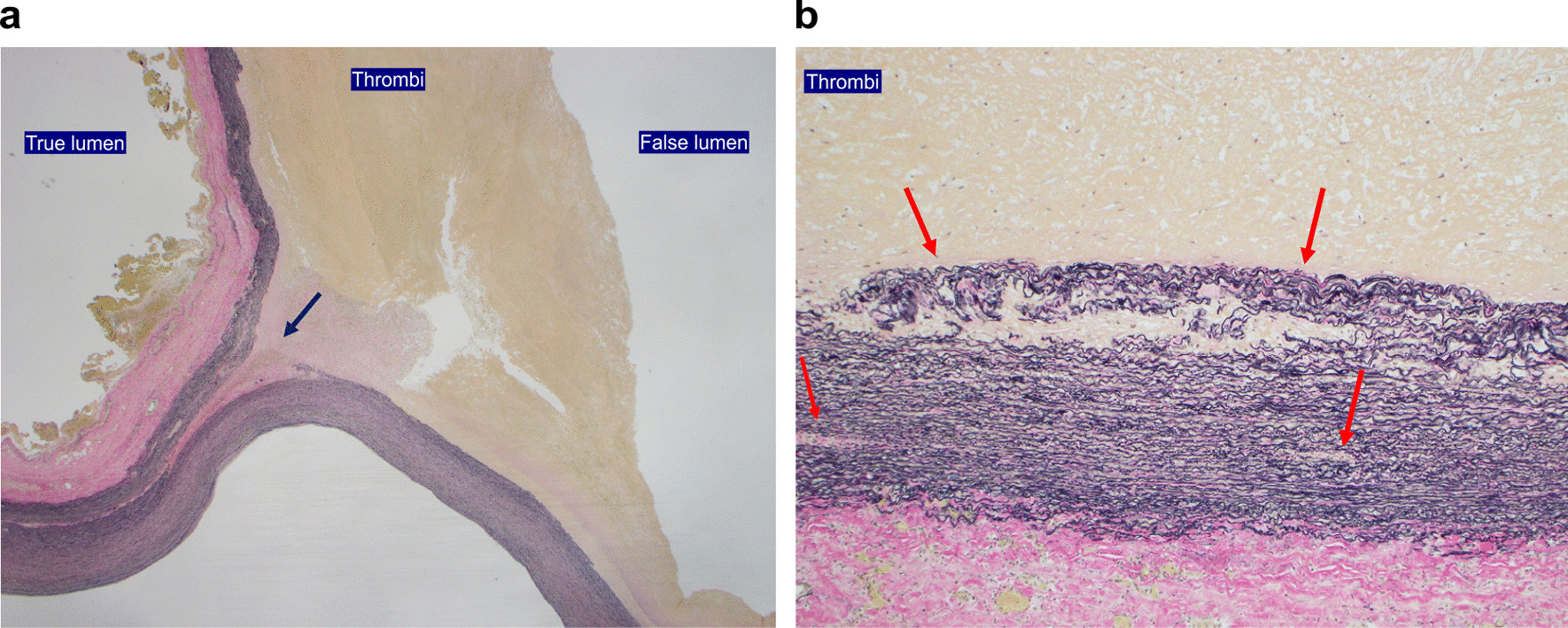
**a**) Pathological examination of the ascending aorta showed medial degeneration with atherosclerosis, fragmentation of media elastica, and aortic dissection. Black arrows indicate the dissection of the media in aortic wall. (**b**) Red arrows indicate fragmentation of elastin in one microscopic field (magnification 100×). Many areas of smooth muscle cell orientation are preserved

**Fig. 6:**
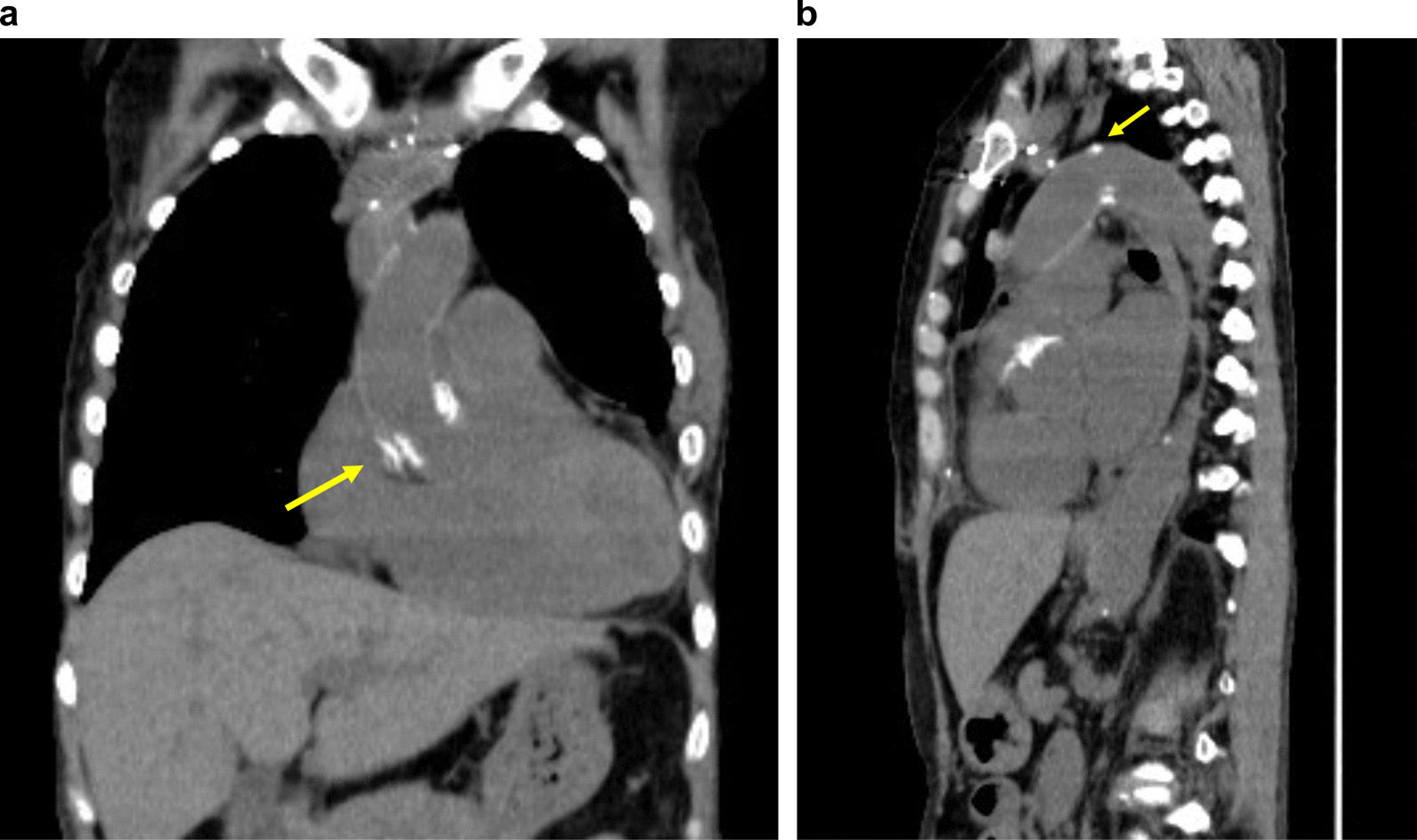
**a**) The white arrow represents the proximal anastomosis. (**b**) The white arrow represents the distal anastomosis

Pathological examination of the ascending aorta revealed aortic dissection with cystic degeneration of the tunica media and fragmentation of the media elastica with atherosclerosis.The operative, CPB, aortic cross-clamp, and circulatory arrest times were 373, 196, 98, and 38 min, respectively. The respiratory support time was 10 h postoperatively. The patient was discharged from the intensive care unit on the second postoperative day, and rehabilitation was initiated. No other complication except atrial fibrillation was noted. CT revealed a well-running prosthetic graft and the absence of pseudoaneurysms at the anastomosis site on both the proximal and distal sides (Figs. 5a and b and 6a and b).

C-reactive protein. decreased gradually, although it did not reach normal values during hospitalization. White blood cell count decreased, thus ruling out infections. Moreover, liver function slowly improved, and albumin recovery was delayed (Table 2).

**Table 2:** Changes in tests during extended aortic arch replacement

	At admission	Day 0	Day 1	Day 2	Day 3	Day 5	Day 10
White blood cells (× 10^9^/L)	10.6	14.0	9.3	10.4	10.8	8.6	9.5
Platelet (×10^4^/µL)	15.3	2.5	9.3	8.7	9.8	7.1	10.9
PT-INR	1.03	1.17	1.17	35.2	1.09	1.10	1.03
aPTT (s)	38.2	33.1	43.5	37.8	34.8	32.0	34.6
AST (U/L)	27	127	93	62	43	40	24
ALT (U/L)	28	26	14	12	8	23	5
Total bilirubin (mg/dL)	0.81	1.35	1.35	1.30	1.35	2.02	1.09
FDP-D (µg/mL)	10.8	-	-	-	9.8		6.9
Total protein (g/dL)	5.7	6.1	4.8	6.0	5.7	7.0	6.5
Albumin (g/dL)	2.9	2.8	2.9	3.5	3.4	3.9	3.6
C-reactive protein (mg/dL)	4.08	0.93	4.8	4.9	3.20	4.04	3.7

*AST* aspartate aminotransferase, *ALT* alanine aminotransferase, *FDP-D* fibrin-fibrinogen degradation product D-dimer, *PT-INR* prothrombin time-international normalized ratio; and *aPTT* activated partial thromboplastin time

Postoperatively, FDP was not elevated. Platelet count recovery was delayed, as it only increased on the 10th postoperative day (Table 2). Moreover, there were no complications related to postoperative thromboembolism or hemorrhage nor the aorta; however, a right-side pyothorax was noted at 4 months postoperatively, a cystic rectal fistula 7 months later, and peritonitis due to colon perforation 1 year later. All complications were treated and resolved immediately. Now, nearly 4 years postoperatively, the patient remains in good health and is being followed up at the outpatient clinic. She has been able to resume her daily activities effectively.

## Discussion and conclusions

With improvements in the prognosis of patients with SLE, aortic disease has gained attention. Wang et al. [2] reported 20 cases of aortic aneurysm and 13 cases of aortic dissection in a retrospective cohort study of 15,209 SLE patients (89.9% females; mean age, 38.3 years) from a Taiwanese population database (overall incidence, 4.26% per 10,000 person-years). Compared to controls, the overall incidence ratio of developing aortic aneurysms or aortic dissection in SLE patients was 3.34 (95% confidence interval, 1.71–6.91; *p* < 0.001). Wang et al. [2] also reported that the mean age of the 21 patients with SLE who developed aortic dissection was 39.3 years and that of those who developed aortic aneurysm was 44.5 years, also highlighting the fact that SLE affects younger individuals [8]. The ratio of women to men with SLE was 10:1. However, the incidence of an aortic aneurysm was significantly higher in men than in women. Women had a substantially higher risk of aortic dissection than men [8]. The etiology of aortic aneurysms and dissections in patients with SLE is diverse and may be complicated by pathological changes, such as atherosclerosis, vasculitis, and medial cystic necrosis in the aorta. Esdaile et al. [1] reported that SLE could cause early atherosclerosis. Prolonged steroid treatment has been shown to accelerate atherosclerosis and hypertension, and aortic histological examination confirms atherosclerosis, degeneration of the aortic elastic tissue, and vasculitis, leading to aortic aneurysm [3]. Marfan-like changes [3] and pathological changes similar to Takayasu’s arteritis have also been reported [9]. Additionally, pathological changes in aortic aneurysms differ depending on their location, with intimal degeneration being more common in the thoracic aorta and atherosclerosis in the abdominal aorta. With the improvement in the prognosis of SLE patients, aortic disease has come into focus. Rapid diagnosis and treatment options for SLE have become available, potentially reducing the progression of aortic aneurysms and other cardiovascular diseases [10, 11]. In addition to the diverse clinical findings, the biochemical and immunological processes should be investigated. Suppression of inflammatory mediators released by inflammatory cells and the neutralization of matrix metalloproteinase(MMP) effects can effectively help inhibit aortic aneurysm growth and dissection [12–14].

Silvestri et al. reported that aortic events are generally more common in women with SLE than in men with SLE and that the estrogen-induced loss of elastic fibers may enhance the vulnerability of the aortic wall caused by the inflammatory burden of SLE [15]. Estrogen inhibits collagen and elastin deposition in the aortic wall, causing loss of the regular arrangement of elastic fibers and hypertrophy or hyperplasia of the smooth muscle vascular cells. It also causes extracellular matrix abnormalities. Conversely, progesterone promotes the deposition of non-collagenous proteins and contributes to the loss of the regular corrugation of elastic fibers. The age at onset of aortic dissection of our patient was 55 years, which is older than that in previous reports. This may be due to accelerated atherosclerosis caused by long-term hypertension and oral steroid use, leading to a degeneration of the tunica media elastic fibers.

In this case, the decision of surgery timing was challenging. Usually, emergency surgery is desirable to treat acute aortic dissection with cardiac tamponade, but it was not possible in this case due to disrupted coagulation caused by liver failure. Surgical treatment proceeded once the patient’s liver function improved. However, the perforation of the small intestine led to peritonitis and bacteremia, which thus required open drainage, partial resection of the small intestine, and the treatment of bacteremia. We decided to perform surgery for aortic dissection after completing this treatment. During the waiting period, the true aortic aneurysm in the distal arch expanded rapidly, prompting total arch replacement surgery in the usual manner. Since there are reports of pseudoaneurysms following aortic dissection surgery [16], and as this patient had SLE, we sutured the aneurysm in the healthy aortic wall.

The endovascular treatment of thoracic aortic aneurysms has attracted attention as an alternative to surgery as it has lower surgical morbidity and mortality than conventional surgery. However, this method also involves several challenges. First, the complication of spinal cord ischemia is not infrequent. Second, we cannot use the endovascular graft when visceral or intercostal artery reconstruction is required. Therefore, the clinical indications of endovascular therapy are limited [17]. Furthermore, the problem of endoleakage persists, even though endovascular treatment is minimally invasive, and there are no publications on the biological response to stents in cases of SLE.

In this case, the main problem was the diagnosis of APS due to positive antiphospholipid antibodies and a history of deep vein thrombosis. Moreover, there was coagulation dysfunction due to severe hepatic injury, and strict perioperative management was undertaken. Previous reports on APS have mentioned finding venous thrombi and medial necrosis in the aortic wall. Thrombosis of the vasa vasorum caused by antiphospholipid antibodies has reportedly caused vulnerability of the vessel wall and impaired blood flow in the vasa vasorum, leading to necrosis of the aortic wall [18–20]. We used a special postoperative management strategy for APS cases or for those with a history and strong suspicion of thrombocytopenia or abnormal coagulation function. We have termed this protocol “the modified triple therapy” (glucocorticoids, anticoagulation, intravenous immunoglobulin, and plasma exchange), a slightly looser version of CAPS treatment [21]. In our case, we administered continuous heparin infusion (10,000–15,000 units/24 h) 5 days before surgery. We also administered IVIG (200 mg/kg/day) for 10 days before and after surgery. Plasma exchange therapy has the following objectives: (1) removal of autoantibodies, (2) removal of other pathogens of large molecular weight, and (3) replenishment of plasma proteins. Tmoo achieve the desired effects, a plasma exchange of at least 1.5 times the normal circulating plasma volume should be achieved. However, we believe that the minimum amount of coagulation factor activity required to expect physiological hemostatic effect and stability of coagulation function after artificial cardioplegia in special cases such as CAPS is approximately 20–30% of the normal circulating plasma volume. Therefore, we exchanged only 30% of the total body plasma volume with FFP during CPB. We recommend administering steroid pulses of 1000 mg of methylprednisolone sodium succinate on the day of surgery and on the day after surgery [22]. In this case, the patient sustained stable coagulation function without the onset of postoperative thromboembolism, and postoperative platelet recovery was good. She was discharged without any other major postoperative complications. In patients with SLE, mesenteric vasculitis causes infarction and perforation of the intestinal wall, especially the small intestine [23]. The immunocompromised state caused by steroid use in SLE can also lead to cytomegalovirus (CMV) infection, causing ulceration and perforation of the intestinal wall [24]. In the present case, a CMV infection was ruled out. Diagnostic imaging, such as chest and abdominal radiography, can miss intestinal perforation in up to 30% of cases; a CT scan is a proper diagnostic method and provides clues on the site of perforation [25].

Although the patient was immunocompromised and hemodynamically unstable, we performed laparotomy due to sepsis. The patient recovered from sepsis, and we were subsequently able to prepare her for aortic dissection surgery. Several previous reports have described cases where immediate surgical intervention was performed for intestinal perforation. In these cases, the patient recovered from sepsis but had an unfavorable outcome due to repeated infections resulting from immunodeficiency [26]. Although our patient suffered repeated intestinal perforations even after surgery for aortic dissection, she recovered from these complications gradually. Even after prescribing steroids and immunosuppressive drugs, abscesses, sepsis, and systemic infections may recur due to decreased immunity. Therefore, in patients with SLE who have previously developed APS, careful follow-up is necessary because local organ ischemia associated with thromboembolism may lead to severe infection.

The strength of this manuscript is that it is well supported by the literature. Moreover, we found the probable cause of the disease and treated it appropriately. As a key limitation of this case report, we could not take preventive measures as we could not adequately manage the condition before the patient developed aortic dissection. Furthermore, we were unable to clarify the relationship between the aortic pathology findings and CAPS.

The evolution of symptoms from the time of onset to admission has been described in this case. The most common initial symptoms of aortic dissection are back and abdominal pains. In this case, the patient continued to rest at home, and the CT scan at the time of admission showed systemic circulatory failure due to cardiac tamponade, accompanied by liver failure. This condition is a significant cause of death in aortic dissection associated with SLE and is extremely dangerous. The patient was treated by a multidisciplinary medical team, including gastroenterologists, gastroenterological surgeons, and collagenologists, which improved her critical condition, and she has since been followed up at the outpatient clinic. In the remote phase, she had also suffered severe complications, which were addressed by information sharing among the medical team members.

In conclusion, systemic circulatory failure due to cardiac tamponade is a significant cause of death in patients with SLE-associated aortic dissection. Although the patient demonstrated many repeated complications, life-threatening complications were avoided because of cooperation and information sharing among the medical team members. A follow-up plan should be established to treat aortic diseases in patients with SLE accompanied by APS.

## Data Availability

The data associated with this manuscript are not publicly available due to privacy and ethical restrictions, but relevant data and images are preserved in the hospital’s medical information system and can be made available by the corresponding author upon reasonable request.

## Abbreviations

SLE: Systemic lupus erythematosus
CT: Computed tomography
APS: Antiphospholipid antibody syndrome
CAPS: catastrophic antiphospholipid syndrome
CPB: Cardiopulmonary bypass
CH50: 50% hemolytic complement activity
FDP: Fibrin-fibrinogen degradation product
FFP: Fresh frozen plasma
IVIG: Intravenous gamma globulin
CMV: Cytomegalovirus
